# Lower plasma 25-hydroxyvitamin D is associated with irregular menstrual cycles in a cross-sectional study

**DOI:** 10.1186/s12958-015-0012-5

**Published:** 2015-03-11

**Authors:** Anne Marie Z Jukic, Anne Z Steiner, Donna D Baird

**Affiliations:** Epidemiology Branch, National Institute of Environmental Health Sciences, PO Box 12233, Durham, USA; Department of Obstetrics and Gynecology, University of North Carolina, Chapel Hill, USA

**Keywords:** Ovulation, Ovary, Vitamin D, Follicle, Menstrual cycle

## Abstract

**Background:**

In animals, low levels of vitamin D are associated with estrus cycle disturbances, but there are virtually no human data. We examined the association of 25-hydroxyvitamin D (25(OH)D) (a biomarker for vitamin D status) with menstrual cycle characteristics.

**Methods:**

Women aged 35-44 were randomly selected from a Washington D.C. health plan and invited to participate in the Uterine Fibroid Study (1996 – 1999). Our analysis includes 636 women (57% were African-American) who provided a blood sample and completed a telephone interview that included gynecologic history. Women were asked their usual cycle length in the preceding year. Women who reported it was “too irregular to estimate” were classified as having irregular cycles (N = 48). Women were excluded if they currently or recently used hormonal contraception or any other medication that influences menstrual cycles. 25(OH)D was measured by radioimmunoassay in stored plasma samples.

**Results:**

The median 25(OH)D level was 12.0 ng/mL (interquartile range: 7.6, 19.7 ng/mL). After controlling for age, race, BMI, education, age of menarche, current smoking, alcohol use, and physical activity, a decrease in 25(OH)D of 10 ng/mL was associated with 1.9 times the odds of irregular cycles (Odds ratio (OR) (95% confidence interval (CI)): 1.9 (1.0, 3.4), p = 0.04). 25(OH)D was not associated with the occurrence of short cycles (OR(CI): 1.08 (0.79, 1.48, p = 0.6) or long cycles (OR(CI): 1.31 (0.66, 2.60), p = 0.4).

**Conclusions:**

Lower levels of 25(OH)D were associated with irregular cycles, but not with short or long cycles. Vitamin D may play a role in regulating ovulatory function. Further investigation of potential mechanisms is warranted.

## Background

Vitamin D is known for its role in bone health [1], but its role in reproduction is a new, active area of investigation [2-4]. Vitamin D receptor is expressed in the ovary, placenta, and the uterus [2-4]. Lower vitamin D has been related to premenstrual syndrome, uterine fibroids, dysmenorrhea and early menarche [4,5].

Diet-induced vitamin D deficiency causes dramatically reduced fertility in both rats and mice, with a 45-70% reduction in the probability of becoming pregnant and a 67-100% reduction in the number of viable pups [2]. Knock-out mice missing the enzyme for converting vitamin D to its active form show estrus cycle disturbances including arrested follicular development, prolonged estrous cycles, and anovulation [6,7].

The promoter region for the gene encoding anti-Müllerian hormone (AMH) contains a domain for the vitamin D response element, suggesting that vitamin D can regulate AMH expression [8]. AMH in turn regulates follicular recruitment, which provides a mechanism for vitamin D to influence ovarian function and menstrual cycle regularity [4]. However, population-based human data regarding vitamin D and menstrual cycles are lacking.

Our objective was to examine the association of vitamin D with menstrual cycle characteristics including long and short cycle length and cycle regularity in a population of randomly-selected members of a prepaid health plan.

## Methods

### Study sample

The National Institute of Environmental Health Sciences (NIEHS) Uterine Fibroid Study, 1996 – 1999, enrolled participants identified from a large health plan in Washington, DC [5,9,10]. In short, randomly selected health plan members between the ages of 30 and 49 were contacted and 80% of those eligible participated (N = 1430). Women completed a telephone administered questionnaire that included reproductive and gynecologic history data. Women were invited to the primary care site for an in-person study visit that included a blood draw. Blood samples were processed and stored at -80°C. For this study, we excluded surgically and naturally menopausal women (N = 189), women who may have used hormonal contraception (N = 102), and women who were not naturally cycling over the index year (N = 53). To further ensure the exclusion of women who were peri-menopausal, we excluded women 45 or over (N = 340). This left 746 women.

All of the women provided informed consent and the study protocol was approved by the NIEHS institutional review board.

### Vitamin D measurement

**Figure 1:**
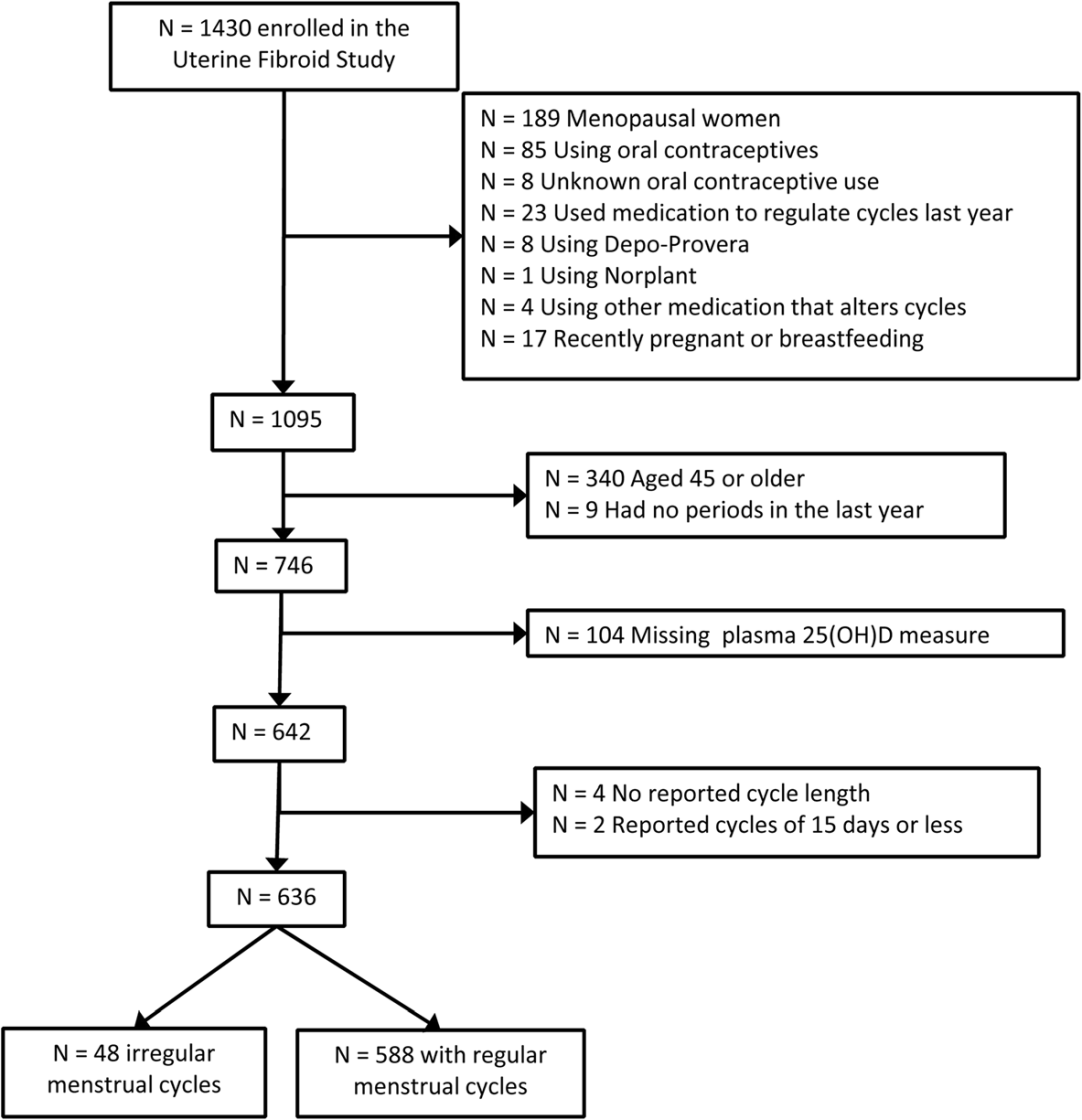
**Flowchart depicting the number of women in the Uterine Fibroid Study included in the analysis of 25(OH)D and menstrual cycle length.**

Vitamin D status was quantified through the measurement of the circulating metabolite 25-hydroxyvitamin D (25(OH)D) in stored plasma samples. 25(OH)D is a widely accepted biomarker for vitamin D [11]. 25(OH)D was measured by radioimmunoassay [12] with a method developed at a laboratory that has been certified by the international Vitamin D External Quality Assessment Scheme, and had been for over 10 years. The antibody was generated by the lab, and was co-specific for both 25(OH)D_2_ and 25(OH)D_3_. The intra- and interassay coefficients of variation based on blind replicates were 7.6% and 10.6%, respectively. One hundred and four women were missing a 25(OH)D measurement, mostly due to a lack of available blood sample, leaving 642 (Figure 1).

### Menstrual cycle characteristics

Women were asked to report their menstrual cycle length in the preceding year. Lower 25(OH)D is hypothesized to interfere with normal follicular development, which might manifest as short, long or irregular menstrual cycles (a combination of normal, short, and long cycles). To investigate this, we separated women into four categories of cycle length: short, long, normal, or irregular. Women who reported that their cycle length was “too irregular to estimate,” and thus could not choose one number to describe their usual cycle length, were classified as having irregular cycles (N = 48). Women who did not report irregular cycles were classified as having “short” cycles if their usual cycle length was 17 - 25 days. We defined “long” cycles as 32-56 days. Given the natural decrease in cycle length among older women, the expected rarity of cycles greater than 35 days [13], and the rarity in our study population, we considered “long” cycles to occur at 32 days or more. The reference category was 26 to 31 days. Four women did not answer the cycle length question and 2 women reported a cycle length of 15 days or less. These women were excluded from the analyses of irregular cycles or cycle length, leaving 636 women (Figure 1).

### Covariates

Data on covariates were collected through self-administered and telephone questionnaires. Body weight was measured at the clinic visit. Potential confounders considered included: age, education, race, body mass index (BMI), alcohol intake, current cigarette smoking, physical activity, and age at menarche.

### Statistical analysis

25(OH)D was structured both as a continuous linear variable and as a dichotomous variable of “insufficient” versus “sufficient” based on the Institute of Medicine cutpoint of 20 ng/mL [14]. Associations between 25(OH)D and short, long and irregular cycles were estimated through multivariable polytomous (also called multinomial) logistic regression to adjust for potential confounders. This models the association between the exposure of interest (25(OH)D) and all of the cycle length categories simultaneously [15]. The regression coefficient from the linear fit to 25(OH)D was multiplied by -10 and then exponentiated to represent the odds of each cycle type for a 10 ng/mL decrease in 25(OH)D. Women who reported regular cycles with a usual cycle length 26 to 31 days served as the referent group. We examined two different adjustment sets. In the first model (“Model 1”), the analysis of 25(OH)D and short, long and irregular cycles was minimally adjusted for age (linear) and race. A second model (“Model 2”) is also presented including adjustment for age and race and in addition, BMI (in four categories), education (dichotomous), alcohol intake (four categories), age of menarche (three categories), current smoking (yes/no), and physical activity (four categories of metabolic equivalent-hours per week). The categories of each covariate are shown in Table 1. An interaction term between 25(OH)D and race was tested in model 2, and was not significant (p = 0.30). We therefore present our results adjusted for race, but not stratified.

**Table 1:** **Characteristics of participants from the Uterine Fibroid Study included in the menstrual cycle characteristics analysis (N = 636), Washington D.C. (1996 – 1999)**

	**N (%)**	**25(OH)D mean (SD)**
25(OH)D		
≤20 ng/mL	484 (76)	--
>20 ng/mL	152 (24)	--
Irregular cycles		
No	588 (92)	14.3 (8.3)
Yes	48 (8)	10.4 (6.1)
Short cycles (17 – 25 days)		
No	453 (71)	14.7 (8.4)
Yes	135 (29)	13.1 (7.9)
Long cycles (32 – 56 days)		
No	566 (89)	14.3 (8.3)
Yes	22 (11)	16.3 (9.4)
Age		
35 - 39	310 (49)	14.4 (8.3)
40 - 44	326 (51)	13.7 (8.1)
Race		
White	216 (34)	20.5 (8.1)
African-American	369 (58)	10.1 (5.8)
Other	51 (8)	15.3 (5.7)
Education		
Lest than a college degree	311 (49)	11.0 (6.5)
College graduate or more	319 (50)	17.0 (8.7)
Missing	6 (0.9)	
Body mass index		
<25 kg/m^2^	258 (41)	17.6 (9.0)
25 - < 30	170 (27)	13.7 (7.5)
30 - <40	159 (25)	10.2 (5.7)
≥40	49 (8)	9.0 (4.5)
Smoker		
Non-smoker	490 (77)	14.8 (8.4)
Current smoker	146 (23)	11.6 (7.1)
Alcohol (drinks per week)		
0	120 (19)	10.6 (6.1)
>0 - 1	188 (30)	13.3 (7.4)
>1 – 7	211 (33)	16.2 (8.5)
>7	79 (12)	15.3 (10.0)
Missing	40 (6)	
Vigorous physical activity		
0 – 1.8 MET-hours/week^a^	203 (32)	11.9 (7.2)
>1.8 – 4.1	203 (32)	14.2 (8.1)
>4.1 – 6.5	108 (17)	15.0 (8.5)
>6.5 – 24.3	121 (19)	16.6 (8.9)
Missing	1 (0.2)	
Age at menarche		
<11 years	51 (8)	12.4 (7.1)
11 - 14	506 (80)	14.3 (8.2)
>14 years	77 (12)	13.4 (8.9)
Missing	2 (0.3)	

^a^MET = Metabolic equivalents.

## Results

Eight percent of study participants reported having irregular cycles, 29% were classified as having short cycles and 11% had long cycles (Table 1). The majority of women were of African-American race and a little over half had at least a college degree. Almost 60% of the women were overweight or obese. The mean 25(OH)D level was 14.0 ng/mL (standard deviation: 8.2), with a median of 12.0 ng/mL (interquartile range: 7.6, 19.7 ng/mL). Most of the women (76%) had insufficient vitamin D concentrations (25(OH)D level below 20 ng/mL).

Lower levels of 25(OH)D were not associated with short or long cycles (Table 2). The overall adjusted association between 25(OH)D and cycle characteristic category, treated as a four-category outcome (typical cycle length, short, long, irregular) was non-significant by a 3 degree-of-freedom likelihood ratio test (p = 0.20). However, in the fully adjusted model, a decrease in 25(OH)D of 10 ng/mL was associated with 1.9 times the odds of having irregular menstrual cycles (Odds ratio (OR) (95% Confidence Interval (CI)): 1.9 (1.0, 3.4), p = 0.04) (Table 2, Model 2). Women who were below the Institute of Medicine recommendation of 20 ng/mL of 25(OH)D had almost two and a half times the odds of having irregular menstrual cycles compared with women who were above 20 ng/mL (OR(CI): 2.4 (0.8, 7.7), p = 0.13). Despite our age restrictions and the self-reported premenopausal status, it is possible that there are perimenopausal women in our sample. To further investigate this, we conducted a sensitivity analysis excluding women who reported taking hormones for menopausal symptoms (N = 5), this did not substantially alter the association between 25(OH)D and irregular cycles (per 10 ng/ml decrease in 25(OH)D, OR(CI): 2.0 (1.1, 3.8), p = 0.03). It is possible that having an intrauterine device (IUD) would affect a participant’s menstrual cycle patterns, though none were using an hormonal IUD. Excluding women with an IUD (N = 14) did not change the estimates (OR(CI): 1.8 (1.0, 3.4), p = 0.05). Excluding women who reported a thyroid disorder did not alter the adjusted estimate (OR(CI): 1.8 (1.0, 3.4)). Twenty-seven women reported a pregnancy in the year prior to interview (N = 15 miscarriages and N = 11 live births) but had menstrual periods at the time of the interview. Also, 28 women reported that they were on medication that regulated their menstrual cycles for “some” of the previous year. We performed a sensitivity analysis excluding these 55 women and saw no difference in the association (OR(CI): 1.9 (1.0, 3.6)). Finally, excluding the one woman who used fertility treatments in the last year (but was never pregnant) did not alter the estimates.

**Table 2:** **Associations of 25-hydroxyvitamin D (25(OH)D) with menstrual cycle characteristics among women from the Uterine Fibroid Study**

	**N**	**Model 1** ^**a**^ **OR (CI) per 10 ng/mL decrease in 25(OH)D**	**p-value**	**N**	**Model 2** ^**b**^ **OR (CI), per 10 ng/ml decrease in 25(OH)D**	**p-value**
Short cycles (≤25 days)	135	1.10 (0.82, 1.49)	0.51	133	1.08 (0.79, 1.48)	0.64
Long cycles (≥32 days)	22	1.28 (0.69, 2.38)	0.43	22	1.31 (0.66, 2.60)	0.44
Irregular cycles	48	2.17 (1.23, 3.83)	0.008	46	1.87 (1.03, 3.39)	0.04
		**Model 1** ^**a**^ **OR(CI) for insufficient versus sufficient 25(OH)D**			**Model 2** ^**b**^ **OR(CI) for insufficient versus sufficient 25(OH)D**	
Short cycles (≤25 days)	135	1.01 (0.60, 1.72)	0.96	133	0.95 (0.55, 1.67)	0.87
Long cycles (≥32 days)	22	1.07 (0.40, 2.84)	0.89	22	1.02 (0.35, 2.96)	0.97
Irregular cycles	48	3.09 (1.02, 9.42)	0.05	46	2.44 (0.77, 7.69)	0.13

^a^Results of the polytomous logistic regression model adjusted for age (linear) and race. The referent category is women who reported regular menstrual cycles and a usual cycle length between 26 and 31 days (N = 431).

^b^Results of the polytomous logistic regression model adjusted for: age, race, BMI, education, age of menarche, current smoking, alcohol use, and physical activity. The referent category is women who reported regular menstrual cycles and a usual cycle length between 26 and 31 days (N = 426).

Only six women reported a prior diagnosis of polycystic ovary syndrome (PCOS). These women tended to have lower 25(OH)D levels (Mean: 6.6 ng/mL vs. 14.1 ng/mL) and the 25(OH)D level for all 6 women was less than 20 ng/ml. It is unclear whether the biology underlying the association of 25(OH)D with PCOS is reflective of the underlying biology that occurs in women without PCOS. Thus, as a sensitivity analysis, we excluded women with PCOS, however the estimated association was unchanged (OR(CI): 1.8 (1.0, 3.3), p = 0.05).

## Discussion

Lower plasma levels of 25(OH)D were associated with an increased odds of having irregular cycles. Plasma 25(OH)D was not associated with the occurrence of long or short menstrual cycles.

25(OH)D is converted to its active form 1,25(OH)D by the enzyme 1α-hydroxylase, coded by the *CYP27B1* gene. *Cyp27b1* null mice and mice that lack the vitamin D receptor have shown hypogonadism, arrested follicular development, prolonged estrous cycles, and hypoplastic uteri [6,7,16,17]. It is unclear whether the reproductive phenotypes in these studies are the result of defects in the ovarian response to gonadotropins or suboptimal gonadotropin secretion from the pituitary or hypothalamus [6]. In *Cyp27b1* null mice the effect of vitamin D deficiency was reversible, with estrous cycle length and staging normalized with dietary supplementation with vitamin D_3_ [6]. Moreover, in this murine model the reproductive effects of vitamin D deficiency appear to occur independent of calcium levels, although this is not conclusive [6,18]. The promoter region for the gene encoding anti-Müllerian hormone (AMH) contains a domain for the vitamin D response element, suggesting that vitamin D can regulate AMH expression [8]. AMH in turn regulates follicular recruitment, which provides some physiological plausibility for vitamin D to influence ovarian function and possibly menstrual cycle regularity [4]. Serum 25(OH)D has been inversely associated with insulin resistance and hyperandrogenism in women with PCOS [4]. Moreover, among women with PCOS, supplementation with vitamin D has been reported to normalize menstrual cycles and improve ovarian folliculogenesis and ovulation [19,20].

Our study is the first to examine the association of vitamin D with menstrual cycle length in a population-based sample of late reproductive-aged women. This analysis is limited by the use of self-reported cycle length and by small numbers of women with extreme cycle lengths, particularly long cycles and irregular cycles. Most of the women in this study had insufficient 25(OH)D levels which, combined with small numbers of irregular cycles, reduced the power to detect an association between the dichotomous 25(OH)D exposure (≤20 ng/ml vs >20 ng/ml) and irregular cycles. This study is based on a cross-sectional design, with women reporting their typical cycle length for the past year at the time of blood collection. It is possible that some of the women in our analysis had undiagnosed PCOS. We did not have other hormonal or clinical markers of PCOS in this sample. Subclinical PCOS might be an underlying factor (or intermediate) in the association of 25(OH)D with irregular cycles.

## Conclusions

We found that lower levels of 25(OH)D were associated with irregular menstrual cycles in a population-based sample of late reproductive-aged women. Vitamin D may influence cycle regularity through its associations with AMH, insulin, androgens, or a yet to be identified pathway. Further investigation of potential mechanisms is warranted.

## Abbreviations

25(OH)D: 25-hyrdoxyvitamin D
PCOS: Polycystic ovary syndrome
NIEHS: National Institute of Environmental Health Sciences
BMI: Body mass index
